# General Practice and Hospital Medicine—A New Relationship

**Published:** 1987-11

**Authors:** Michael Whitfield

**Affiliations:** Senior Lecturer in General Practice, Dept of Epidemiology and Community Medicine, University of Bristol


### General Practice and Hospital Medicine—a new relationship?

**Michael Whitfield MA, MB, B.Chir. FRCGP.**
Senior Lecturer in General Practice,
Dept of Epidemiology and Community Medicine, University of Bristol

I have recently been looking through questionnaires completed by fifth year medical students after they have completed 2 week attachments to general practices in the South West. They are usually resident with the doctor and – often for the first time in their life – have the opportunity of a one to one relationship with a doctor for an extended period of time. One of the questions I ask the students to address is "Has this attachment altered your previous view about hospital medicine?"

Comments are very varied but included the following: *The attitude of SHOs to GPs letters seems a touch arrogant on occasions considering they have no background knowledge of patients. Reinforced the need for good communication between hospital and GP. How important it is for hospital staff not to make disparaging remarks about the patients' GP's skills in the presence of the patient. Just as some GPs are blacklisted by some consultants as being appalling, poor communicators etc, the reverse seems to apply. GPs are not the babbling duffers they seem – consultants are good, but only in certain areas. I don't want to be a hospital doctor! Junior doctors in hospital assume GPs are guessing. The GP undergoes a clinical viva before obtaining an admission. The impersonal nature of hospitals was emphasised by the personal care of the GPs. Spending time with a GP helps one to realise how many cases presenting to a doctor are dealt with outside hospital. It also helps one to realise that often voiced complaints by hospital doctors about GPs could with justice be reciprocated by GPs.*

Many of these students are going to see no more general practice at first hand in their careers. They will go on to become specialists. Their whole attitude to general practice will have been coloured by their experiences as medical students and they will go on perpetuating the ideas described above. That many of the students have gained insight and learnt about the benefits of good general practice pleases me; but I am bothered by the fact that some of the others will continue in their careers to propogate the traditional teaching hospital attitudes.

Let's leave this worrying area and examine other areas where general practice and hospital medicine need a new relationship. I am going to look at four areas;

Referrals
Hospital followup
GP hospital beds
Community Care

General practitioners are expected to refer patients to specialists for various purposes: for diagnostic help, for technical help, for example, performing an operation, and sometimes to share a problem. General practitioners, as you will have heard already do this with varying degrees of approval. Not only approval though – they do this with varying degrees of enthusiasm! Some general practitioners refer as infrequently as twice in every 100 consultations, others as frequently as 25 in every 100 consultations. The mean is 8 in 100 consultations. Why is there this large variation? Unfortunately we do not know. We have examined the likely factors: it is not the site of the practice, nor the age of the doctor or his gender. His medical school doesn't determine his rate nor his qualifications. It seems that each general practitioner has a unique 'referral threshold' that varies within practices so that one partner will be always referring and another rarely. When are these referral patterns learnt? Is it personality that influences our referral rates? .... we just do not know.

Whatever the factors we know that there are consequences from this referral process. We know that each outpatient appointment costs the NHS about £20. This is, of course the minimum. If a patient is admitted or investigated the costs rise astronomically. So each referral can result in incredible cost consequences for the NHS. The surprising fact is that on-one in the NHS appears to be studying the variation; we have no mechanism for doing this; we cannot even feed back to general practitioners the simple information about how many referrals they are making and how they compare with their colleagues. Hospitals have no mechanism for recording which general practitioner refers a patient to outpatient departments on their computer systems; it is a low priority!

Consultants frequently feel overwhelmed by referrals and react in a number of ways; some use their junior staff and compound the problems of cost as it is well known that juniors rely on investigations more than their seniors; to say nothing of their lack of experience.

Others allow the waiting lists to grow and hope that general practitioners will be put off referring by a two year waiting list for 'routine problems'. There is some justification for this; I'm sure it works, but it doesn't give the NHS a very good press. One other reaction to this pressure of referrals is to create an empire. I'm sure that you have all seen the way some specialties have grown over the last few years. Patients are never discharged; waiting lists grow; the clinics get longer and impinge on other specialities and the administration is shown the need for another consultant and supporting team. This problem of waiting lists and referrals needs to be reexamined.

Several years ago I asked a group of newly qualified consultants whether they agreed with the statement that 'consultants followup too many patients in outpatients'. The great majority agreed with me. The reasons this occurs are many and include the consultants' natural wish to see the results of treatment and advise. Other reasons include the wish to maintain a register of patients for teaching and research and the feeling, often expressed, that the patient's disease cannot be trusted to the general practitioner. Very often, though, the followup is done by a very junior member of staff and the rationale for the procedure becomes distinctly questionable. This increasing tendency to follow up patients in outpatient departments can only lead to a deterioration in the relationship between consultant and general practitioner. The former will continue to undervalue the latters' skills and the general practitioners' skills will continue to atrophy.

An alternative, and more economic procedure is to refer the patient back to the patient's general practitioner as soon as possible – ideally after an opinion has been expressed or a procedure has been performed.

I and my colleagues in the health centre are privileged in having direct access to both obstetric and general hospital beds. We are able to admit suitable patients to each and contract to provide 24 hour cover for these patients. We are able to admit patients for terminal care, patients with conditions like respiratory infections, the elderly with myocardial infarction and so on. This privilege is not shared with all general practitioners; many would not want it! It certainly causes us more work but it is certainly satisfying both to us and the patients. Why do not more health authorities provide this type of 'low tech' admission facility for general practitioner care? It certainly is less expensive than the normal hospital bed, is satisfying to both patient and doctor and takes from the specialist physician patients who do not merit his expertise.

General practitioners offer care to individuals and increasingly to the community. Unfortunately, as the history of general practice shows there is a tendency for others to attempt to provide community care as well. Antenatal clinics used to be run by the Local Health Authorities. These authorities still run Family Planning clinics and Well Baby Clinics. The hospital specialities are beginning to stretch out into the community and employ psychiatric community nurses, geriatric, diabetic and premature baby health visitors and so on. Why is this occuring? Is it to help general practitioners care for their patients? The usual reason, I suspect is that the hospital authorities are developing an outreaching ambition, partly to extend an empire but partly because they do not understand the role of the general practitioner.

I started off talking about a new relationship between hospital and general practice and have ended on a critical note. I would not like to do this as I see the need to create bridges and understand not antagonism. The four areas of difficulty I have described are four areas of difficulty for both hospital practice and general practice. We are both at fault for the difficulties and we can both strive for an improvement in the relationship. Certainly from the viewpoint of a final year medical student in Bristol, we need to improve.

*If hospital doctors have their favourite stories about incompetent GPs then GPs certainly have many a horrific tale regarding their hospital colleagues. Neither are perfect but with an almost pure diet of hospital medicine until now I was certainly biassed in favour of its ivory towers. It always helps to view things from different angles.*

*Paper delivered to Bristol Medico– Chirurgical Society on the occasion of their visit to the Whiteladies Health Centre, 11th March 1987.*

